# Impact of COVID-19 pandemic on management of autoimmune and inflammatory diseases in Morocco

**DOI:** 10.11604/pamj.2020.37.240.26188

**Published:** 2020-11-16

**Authors:** Ammouri Wafa, Harmouche Hicham, Khibri Hajar, Maamar Mouna, Mezalek Tazi Zoubida, Adnaoui Mohamed

**Affiliations:** 1 Internal Medicine Department, Ibn Sina Hospital, Rabat, Morocco,; 2 Faculty of Medicine and Pharmacy, Mohammed V University, Rabat, Morocco

**Keywords:** Autoimmune, inflammatory, disease, COVID-19, pandemic

## To the editors of the Pan African Medical Journal

The new coronavirus disease (COVID-19) pandemic originated from Wuhan, China, at the end of 2019 [1] and has now rapidly spread over the world. This pandemic is the defining global health crisis of our time and the greatest threat we have faced during this century. As a highly contagious virus, the infections rapidly spread globally; with the most affected regions being the USA, South-East Asia, Europe and Eastern Mediterranean. The African continent is less affected but a gradual increase in cases has been noted since two months ago in several African countries including Morocco. According to the World Health Organizations' (WHO) data, in globally, in 19^th^ September 2020, there have been 30,369,778 confirmed cases of COVID-19, including 948,795 deaths [2]. To date, in the absence of any specific treatment, our knowledge of the disease is better known but is subject to rapid change. This has led to the prompt development of clinical patient risk stratification tools to aid in determining the need for testing, isolation, monitoring, ventilator support and disposition.

Individuals with autoimmune or inflammatory diseases (AI/ID) may require special consideration because receiving immune-suppressive therapies, made them more susceptible to viral and bacterial infections. COVID-19 infection may represent a serious danger for patients with AI/ID due to their immunocompromised state [3]. These patients may be at higher risk for a more severe course with COVID-19, including hospitalization, complications and death. To date, it has not shown a higher incidence of COVID-19 infection in patients with rheumatic diseases compared to the general population [4,5]. The prevalence of hospitalizations due to COVID-19 infection is most reported in systemic autoimmune or immune-mediated disease. It is remarkable the relatively low rate in patients with systemic lupus erythematosus, despite an expected greater use of corticosteroids and immune-suppressants. A possible explanation is the frequent use of anti-malarials, which might have played a protective role as proposed according to their in vitro antiviral effect. Also, patients treated with anti-rheumatic drug (b-DMARD) therapy or non-steroidal anti-inflammatory drug (NSAID) and infected with COVID-19 did not develop life-threatening complications due to their underlying medication [4]. This apparently surprising finding can better be explained through the comprehension of the pathological mechanisms leading to acute respiratory distress syndrome, in which overexpression of inflammatory mediators plays a crucial role. However, glucocorticoid exposure of ≥10 mg/day in patients with AI/ID is associated with a higher odd of hospitalization. While biological therapies and antimalarial drugs like hydroxychloroquine (HCQ) were not associated with a higher risk of hospitalization for COVID-19. A high lethality is reported among elderly patients, with high rheumatic disease activity and/or several comorbidities such as: dyslipidemia, cardiovascular disease and interstitial lung disease [5,6].

In addition to the virulence of the infection, the medical management of AI/ID during a pandemic has become complex. The restrictive government measures cause many obstacles and difficulties for access to hospitals with population confinement. Drug manufacturing and available medicines are compromised. However, depending on the pandemic level and the healthcare system in each country, hospital beds might be lacking. To overcome this crisis, some recommendations and emergency measures have been developed by scientific societies like the European League Against Rheumatism (EULAR) and the American College of Rheumatology (ACR) [7,8]. Their guidelines are established according to the coronavirus high contagion rate in Europe. Currently, virus prevention procedures remain very useful means to avoid infection and the WHO recommendations must be diligently followed by healthcare staff and patients. It is vital to be very careful with hygiene routines, including hand washing, use of protective masks, alcohol-containing hand sanitizers and limited visits. For stable patients with low disease activity it is recommended to prioritize telemedicine consultations and web-based triage if possible and we support the approach of maintaining, chronic immune-suppressants or biologics treatments and the lowest dose of glucocorticoids.

In Morocco, the situation is still critical since two months ago, with a record of 99.816 confirmed cases with 1795 deaths [9]. The continued challenges of social distancing, containment, isolation and surge capacity in already stressed hospitals, clinics and emergency departments. The government has adopted containment measures very early but the contagion is still gaining ground and to ensure the safety of our patients with AI/ID, our internal medicine department centres have adopted actions based on the European recommendations. Also, we have recently available preliminary recommendations of African League against Rheumatism (AFLAR) made by rheumatologists, which are valid for AI/ID followed in internal medicine [10]. The most important message is the postponement, it may be cautiously recommended to comply with all preventive and control measures prescribed by the health authorities in their countries. Also, it is cautiously recommended to continue glucocorticoids and other disease-modifying anti-rheumatic drugs (DMARDs) in patients receiving these therapies, with discontinuation of DMARDs during infections as per standard practice. Regarding the HCQ treatment, as the drug is used in the protocol of treatment of COVID-19 in Morocco, it is necessary to reserve the full-dose prescription of this medication for pathologies for which the indication is approved (e.g. systemic lupus erythematosus (SLE) patients) in order to avoid over-prescription of HCQ and drug shortage. In Table 1, we summarize the main practical steps that can be taken to reduce the risk to our vulnerable patients with AI/ID.

**Table 1:** practical recommendations for management of autoimmune or inflammatory diseases during COVID-19 pandemic

Hygiene procedures management of all autoimmune or inflammatory disease	Patients should be counselled on general preventive measures, e.g. social distancing, hand hygiene, wearing masks in public places
	Reduce the frequency of routine laboratory surveillance when the associated risk of not testing is deemed to be low. Optimal use of tele-health
	Patients should have their vaccination against influenza and pneumococcal updated
	Anti-malarials such as HCQ and CQ do not protect against SARS-CoV-2 and as such, patients on HCQ or CQ for AI/ID should be advised to observe necessary precautions to prevent infection
	Where possible, the use of subcutaneous formulations of bDMARDs and bsDMARDs should be considered instead of IV infusions to limit patients' attendance to the hospital
	Continued use of ACE inhibitors and ARBs per standard of care in rheumatic disease (patient with a history or risk of scleroderma renal crisis or those with SLE and hypertension)
	Possibility to potential temporary delays in performing intravenous administration of zoledronic acid or subcutaneous administration of denosumab: dosing intervals with denosumab not exceed 8 months due to concerns regarding increased vertebral fracture risk following denosumab withdrawal
Drugs for All autoimmune or inflammatory disease	Glucocorticoids should be used at the lowest dose possible to control rheumatic disease, regardless of exposure or infection status
	Glucocorticoids should not be abruptly stopped, regardless of exposure or infection status
	HCQ/CQ, SSZ, MTX, LEF, immune-suppressants (e.g. tacrolimus, CSA, MMF, AZA), biologics (TNF or IL- 6 receptor inhibitors), JAK inhibitors and NSAIDs may be continued
	In the context of a drug shortage due to COVID-19, new HCQ/CQ prescriptions for non-approved indications should be avoided
	ACE inhibitors or ARBs should be continued in full doses or initiated
Systemic lupus erythematosus patients	Newly diagnosed disease: HCQ/CQ should be started at full dose and must be continued during pregnancy
	For patients with organ-threatening disease nephritis or vasculitis), high-dose glucocorticoids or immune-suppressants (e.g. tacrolimus, CSA, MMF, AZA) may be initiated
Following SARS-CoV-2 exposure	HCQ/CQ, SSZ, and NSAIDs may be continued. Immuno-suppressants (e.g. tacrolimus, CSA, MMF, AZA), non-IL-6 biologics, and JAK inhibitors should be stopped temporarily, pending a negative test result for COVID-19 or after 2 weeks of symptom-free observation
Re-iniating treatment following COVID-19	Decisions regarding the timing of reinitiating rheumatic disease therapies in patients recovering from severe COVID-19 related illness should be made on a case-by- case basis
	For patients with uncomplicated COVID- 19 infections: restarting rheumatic disease treatments within 7-14 days of symptom resolution
	For patients who have a positive PCR test result for SARS-CoV-2 but are (and remain) asymptomatic, consideration may be given to restarting rheumatic disease treatments 10-17 days after the PCR result is reported as positive

DMARD: disease-modifying anti-rheumatic drug; SARS-CoV-2: severe acute respiratory syndrome coronavirus 2; NSAID: non-steroidal anti-inflammatory drug; COVID-19: coronavirus disease 2019; HCQ: hydroxychloroquine; CQ: chloroquine; csDMARD: conventional synthetic disease-modifying anti-rheumatic drug; bDMARD: biologic disease-modifying anti-rheumatic drug; bsDMARD: biosimilar disease-modifying anti-rheumatic drug; ACEI: angiotensin-converting enzyme inhibitor; ARB: angiotensin receptor blocker; PCR: polymerase chain reaction; AI/ID: autoimmune/inflammatory diseases; rheumatic disease treatments: (e.g. DMARDs, immunosuppressants, biologics and JAK inhibitors); SSZ=sulfasalazine; MTX= methotrexate; LEF= leflunomide; CSA= cyclosporin A; MMF= mycophenolate mofetil; AZA= azathioprine; IL-6= interleukin-6

## Conclusion

To date, there is no evidence of a higher incidence of COVID-19 in patients with auto-immune rheumatic diseases but rather have a small increased risk of developing severe forms. However, a series of cases demonstrates that the majority of patients with AI/ID or on immunosuppressive therapies captured in different registries recover from COVID-19, which should provide some reassurance to patients. The preventive role of HCQ or conventional synthetic, biological or targeted synthetic drugs remains to be demonstrated. It is necessary to carefully follow all international recommendations. Morocco has therefore taken emergency measures to minimize the impact of COVID-19 on the management of patients with AI/ID.
